# Mass-spectrometry analysis of histone post-translational modifications in pathology tissue using the PAT-H-MS approach

**DOI:** 10.1016/j.dib.2016.02.028

**Published:** 2016-02-16

**Authors:** Roberta Noberini, Giancarlo Pruneri, Saverio Minucci, Tiziana Bonaldi

**Affiliations:** a Center for Genomic Science of IIT@SEMM, Istituto Italiano di Tecnologia, Via Adamello 16, 20139 Milan, Italy; b School of Medicine, University of Milan, 20122 Milan, Italy; c Biobank for Translational Medicine Unit, Department of Pathology, European Institute of Oncology, Via Ripamonti 435, 20141 Milan, Italy; d Department of Experimental Oncology, European Institute of Oncology, Via Adamello 16, 20139 Milan, Italy; e Drug Development Program, European Institute of Oncology, Via Adamello 16, 20139 Milan, Italy; f Department of Bioscience, University of Milan, 20133 Milan, Italy

**Keywords:** Mass spectrometry, Proteomics, Epigenetics, Histone posttranslational modifications, Formalin-fixed paraffin embedded tissues, Biomarker, Breast cancer

## Abstract

Aberrant histone post-translational modifications (hPTMs) have been implicated with various pathologies, including cancer, and may represent useful epigenetic biomarkers. The data described here provide a mass spectrometry-based quantitative analysis of hPTMs from formalin-fixed paraffin-embedded (FFPE) tissues, from which histones were extracted through the recently developed PAT-H-MS method. First, we analyzed FFPE samples from mouse spleen and liver or human breast cancer up to six years old, together with their corresponding fresh frozen tissue. We then combined the PAT-H-MS approach with a histone-focused version of the super-SILAC strategy-using a mix of histones from four breast cancer cell lines as a spike-in standard- to accurately quantify hPTMs from breast cancer specimens belonging to different subtypes. The data, which are associated with a recent publication (Pathology tissue-quantitative mass spectrometry analysis to profile histone post-translational modification patterns in patient samples (Noberini, 2015) [1]), are deposited at the ProteomeXchange Consortium via the PRIDE partner repository with the dataset identifier PXD002669.

**Specifications table**

**Table:** 

Subject area	*Biology*
More specific subject area	*Proteomics*
Type of data	*Mass spectrometry raw data and MaxQuant search results*
How data was acquired	*Data dependent LC-MS/MS acquired on a Q Exactive instrument (Thermo Fisher Scientific) coupled to a ultra nanoflow high-performance liquid chromatography (HPLC) system (EASY-nLC™ 1000, Thermo Fisher Scientific)*
Data format	*MS-RAW files, experimental design and.txt and.apl output files generated by the MaxQuant software*
Experimental factors	*Histones were purified from frozen and FFPE mouse and human samples*
Experimental features	*Purified/enriched histones were in-gel digested through an Arg-C-like digestion and subjected to LC/MS/MS analysis. A heavy-labelled histone spike-in standard was used to quantitate hPTMs in human samples*
Data source location	*Milan, Italy, European Institute of Oncology*
Data accessibility	*Data have been deposited to the ProteomeXchange Consortium via the PRIDE partner repository with the dataset identifier PXD002669*

**Value of the data**

• *Analysis of hPTM marks in FFPE tissue of different origin (mouse spleen and liver and human breast cancer biopsies) and their corresponding fresh-frozen tissues.*
• *A histone-focused version of the super-SILAC approach used to accurately quantitate hPTMs in breast cancer samples.*
• *Quantitative analysis of hPTMs in 20 breast cancer samples belonging to different subtypes, whose immunoprofile and staging are provided*
• *The data can be used to profile less common PTMs, such as K-ubiquitylation, R-methylation, K-crotonylation, as well as histone H3 variants, among different cancer subtypes.*

## 1 Data

**Fig. 1:**
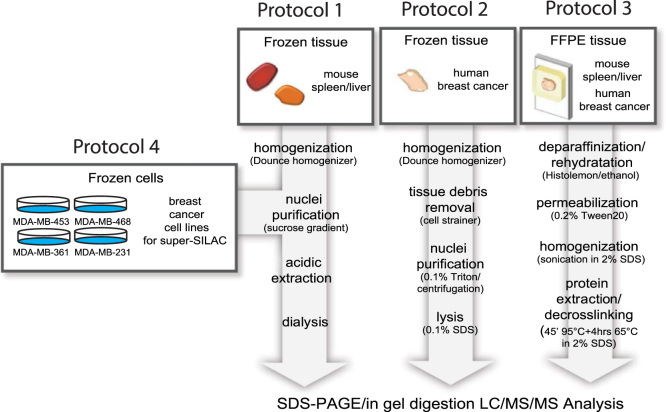
Scheme of protocols used to isolate histones from different types of samples. Protocol 1: Frozen mouse liver and spleen. Protocol 2: Frozen human breast cancer tissue. Protocol 1: FFPE tissues (mouse and human). Protocol 4: Heavy-labeled breast cancer cell lines for super-SILAC mix.

This proteomics dataset comprise LC-MS/MS raw files obtained from bottom-up MS analysis of histone H3 and H4 isolated using different procedures (Fig. 1) from mouse and human tissues, which were either stored as frozen samples or formalin-fixed and paraffin-embedded (FFPE). The dataset also includes the output files of the database search for common hPTMs and formalin-induced modifications.

## 2 Experimental design, materials and methods

The data correspond to three main experiments (Tables 1 and S1): (1) analysis of FFPE and corresponding frozen tissue in mouse spleen or liver, stored for few weeks up to 6 years, (2) analysis of FFPE and corresponding frozen tissue in breast cancer samples derived from 3 patients, and (3) analysis of 20 FFPE breast cancer tissues belonging to different subtypes (Luminal A-like, Luminal B-like, Triple Negative and HER2 positive). The analysis of breast cancer samples was carried out using a super-SILAC mix of heavy-labeled histones from 4 breast cancer cell lines as internal standard.

### 2.1 Preparation of fresh-frozen and FFPE tissues

Spleen tissue was obtained from leukemic mice as previously described [2] and divided into two portions. One portion was washed and homogenized in ice-cold phosphate buffered saline (PBS: 0.8% NaCl; 0.02% KCl; 0.02% KH_2_PO_4_; 0.2% Na_2_HPO_4_, pH 7.4) using a Dounce homogenizer to obtain spleen cells that were counted, pelleted by centrifugation, frozen and stored at −80 °C until use. The other half of the spleen was rapidly washed in PBS and incubated for 16 h at room temperature in 4% paraformaldehyde. The fixed spleen was dehydrated with increasing concentrations of ethanol (70%, 80%, 90% and 100%) and then included in paraffin using a tissue processor (Leica ASP300). Frozen cells and FFPE samples were prepared similarly from mouse liver. Experimental procedures involving animals complied with the National Institutes of Health guide for the care and use of Laboratory animals (NIH Publications no. 8023, revised 1978), and were approved by the Institutional Ethical Committee.

Breast cancer specimens were obtained from 23 patients with duct invasive carcinoma not otherwise specified (Table 2), who were subjected to mastectomy or breast conserving surgery. The patients provided informed consent and this study was approved by the Ethical Committee of the European Institute of Oncology. Tumor samples were collected and snap frozen or fixed 4% formalin and embedded in paraffin. The immunoprofile of the samples was determined by immunohistochemistry, using the anti-ER/PgR (PharmDX, Dako), the Her-2 (Herceptest, Dako) and the anti-Ki-67 antibody MIB-1. Breast cancer subtypes were defined according to the 2013 S. Gallen consensus conference recommendations: Luminal A-like: ER and/or PgR(+), HER2(−), Ki67<20%; Luminal B-like: ER and/or PgR(+), HER2(−), Ki67≥20; Triple Negative: ER, PgR and HER2(−), irrespective of Ki67 score; HER2-positive: HER2(+), irrespective of ER, PgR or Ki67. ER/PgR positivity was defined as≥1% of immunoreactive neoplastic cells; HER2 positivity was defined as>10% of neoplastic cells with strong and continuous staining of the cell membrane (3+ by immunohistochemistry) and/or amplified by in situ hybridization techniques.

### 2.2 Histone isolation from fresh-frozen mouse spleen and liver tissues (Fig. 1, Protocol 1)

Cells were obtained from fresh mouse spleen and liver as described above and histones were isolated as previously described [3]. Briefly, 30×10^6^ cells were resuspended in lysis buffer (10% sucrose; 0.5 mM EGTA, pH 8.0; 15 mM NaCl; 60 mM KCl; 15 mM HEPES; 0.5% Triton; 0.5 mM PMSF; 1 mM DTT; 5 mM NaF; 5 mM Na_3_VO_4_; 5 mM Na-butyrate; protease inhibitors) and nuclei were isolated by centrifugation on a sucrose cushion. Histones were extracted by overnight incubation in 0.4 N HCl at 4 °C and dialyzed against 100 mM CH_3_COOH, using dialysis membranes with a 6–8 kDa cutoff (Spectrumlab). The dialyzed samples were lyophilized and stored at −80 °C.

### 2.3 Histone isolation from fresh-frozen human breast cancer tissues (Fig. 1, protocol 2)

20–70 mg of frozen breast cancer tissue were thawed on ice, cut in small pieces with scissors and then homogenized with a Dounce homogenizer in PBS containing 0.1% Triton X-100 and protease inhibitors. Tissue debris were removed by filtration through a 100 μm cell strainer and nuclei were isolated through a 10 min centrifugation at 2300×g. Nuclei were resuspended in 100–200 μl of the same buffer including 0.1% SDS and incubated with 250 U of benzonase (Merk Millipore) for few minutes at 37 °C to digest nucleic acids. Histone concentration and purity was assessed using the Bradford protein assay kit (Thermo Fisher Scientific) and SDS-PAGE.

### 2.4 Histone isolation from FFPE tissues (PAT-H-MS, Fig. 1, protocol 3)

Four 10-μm tissue sections were deparaffinized by repeating 5 times addition of 1 ml of hystolemon (Dasit Group Carlo Erba), vortexing for 30 s and centrifuging at 18,000×g for 3 min. The samples were then rehydrated in decreasing concentrations of ethanol (100%, 95%, 70%, 50%, 20% and water) for 3 min at room temperature, followed by a 3−5 min centrifugation at 18,000×g. Rehydrated FFPE sections were permeabilized in 0.5 ml of Tris-buffered saline (TBS) containing protease inhibitors and 0.5% Tween20 for 20 min at room temperature in a rotating platform, followed by a 5 min centrifugation at 18,000×g. The samples were resuspended in 200 μL of 20 mM Tris pH 7.4 containing 2% SDS and sonicated in a Branson Digital Sonifier 250 with a 3 mm microtip until all tissues pieces were homogenized. Proteins were extracted and de-crosslinked with a 45 min incubation at 95 °C, followed by a 4 h incubation at 65 °C. After estimating the protein concentration with the Biorad DC protein assay kit (Biorad), 16–20 μg of proteins were run on a 17% SDS-PAGE gel together with known amounts of recombinant histone H3.1, which was used as a standard to estimate histone concentration.

### 2.5 Histone isolation from breast cancer cell lines (Fig. 1**,** Protocol 4)

MDA-MB-231, MDA-MB-468, MDA-MB-453 and MDA-MB-361 (ATCC) were grown in SILAC-DMEM (Euroclone) supplemented with 2 mM L-glutamine, 146 mg/l of lysine (Sigma-Aldrich), 84 mg/l L-^13^C_6_^15^N_4_-arginine (Arg-10, Sigma-Aldrich), 10% dialyzed serum (Life Technologies) and penicillin/streptomycin for approximately 8 doublings to obtain complete labeling. Histones were isolated using the same procedure described for cells obtained from fresh-frozen mouse tissues. Equal amounts of histones from each cell line were mixed, aliquoted, lyophilized and stored at −80 °C until use.

### 2.6 Histone digestion

Approximately 4–5 μg of histones per run per sample were separated on a 17% SDS-PAGE gel and bands corresponding to histones H3 and H4 were excised and in-gel digested as previously described [1], [3]. Briefly, gel bands were cut in pieces, destained and in-gel chemically alkylated with D_6_-acetic anhydride (Sigma-Aldrich) 1:9 in 1 M NH_4_HCO_3_, using CH_3_COONa as catalyzer. After a 3 h incubation at 37 °C, the gel slices were washed with NH_4_HCO_3_, followed by ACN at increasing percentages (from 50 to 100). The in-gel digestion was performed overnight with 100 ng/μL trypsin (Promega) in 50 mM NH_4_HCO_3_ at 37 °C, in order to obtain an “Arg-C like” in-gel digestion. Digested peptides were extracted using 5% formic acid alternated with ACN 100%. In SILAC experimental set-ups, unlabeled and heavy-labeled histones were mixed in equal amounts prior to gel separation, and then processed as described above. Digested peptides were desalted and concentrated using a combination of reversed-phase C_18_/C and strong cation exchange (SCX) chromatography on handmade nanocolumns (StageTips). Digested peptides were then eluted with 80% ACN/0.5% acetic acid and 5% NH_4_OH/30% methanol from C_18_/C and SCX StageTips, respectively. Eluted peptides were lyophilized, resuspended in 1% TFA, pooled and subjected to LC-MS/MS analysis.

### 2.7 LC-MS/MS

Peptide mixtures were separated by reversed-phase chromatography on an in-house-made 25 cm column (outer diameter 350 μm, inner diameter 75 μm, 1.9 μm ReproSil, Pur C18AQ medium), using a ultra nanoflow high-performance liquid chromatography (HPLC) system (EASY-nLC™ 1000, Thermo Fisher Scientic) connected online to a Q Exactive instrument (Thermo Fisher Scientific) through a nanoelectrospray ion source. Solvent A was 0.1% formic acid (FA) in ddH_2_O and solvent B was 80% ACN plus 0.1% FA. Peptides were injected in an aqueous 1% TFA solution at a flow rate of 500 nl/min and were separated with a 100 min linear gradient of 0–40% solvent B, followed by a 5 min gradient of 40–60% and a 5 min gradient of 60–95% at a flow rate of 250 nl/min. The Q Exactive instrument was operated in the data-dependent acquisition (DDA) mode to automatically switch between full scan MS and MS/MS acquisition. Survey full scan MS spectra (*m*/*z* 300–1650) were analyzed in the Orbitrap detector with resolution of 35,000 at *m*/*z* 400. The five most intense peptide ions with charge states≥2 were sequentially isolated to a target value for MS1 of 3×10^6^ and fragmented by HCD with a normalized collision energy setting of 25%. The maximum allowed ion accumulation times were 20 ms for full scans and 50 ms for MS/MS and the target value for MS/MS was set to 1×10^6^. The dynamic exclusion time was set to 20 s and the standard mass spectrometric conditions for all experiments were as follows: spray voltage of 2.4 kV, no sheath and auxiliary gas flow.

### 2.8 Data analysis

Acquired RAW data were analyzed by the MaxQuant software v.1.3.0.5 with the Andromeda search engine [4]. The Uniprot MOUSE 1301 and HUMAN 1301 databases were used for peptide identification. Enzyme specificity was set to Arg-C. The estimated false discovery rate of all peptide identifications was set at a maximum of 1%. The mass tolerance was set to 6 ppm for precursor and fragment ions. Three missed cleavages were allowed, and the minimum peptide length was set to 6 amino acids. Variable modifications were lysine D_3_-acetylation (+45.0294 Da), lysine monomethylation (+59.0454 Da, corresponding to the sum of D_3_-acetylation (+45.0294) and monomethylation (+14.016)), dimethylation (+28.031 Da), trimethylation (+42.046 Da), and lysine acetylation (+42.010 Da). The raw data were analyzed through multiple parallel MaxQuant jobs, setting different combinations of variable modifications: (1) D_3_-acetylation, lysine monomethylation with D_3_-acetylation, dimethylation and lysine acetylation, (2) D_3_-acetylation, lysine monomethylation with D_3_-acetylation, dimethylation and trimethylation, (3) D_3_-acetylation, lysine monomethylation with D_3_-acetylation, trimethylation and lysine acetylation (4, only for mouse samples) D_3_-acetylation, formylation (+27.9949 Da), methylene adducts (+12 Da) and methylol adducts (+30.0106 Da). For SILAC experiments, Arg10 was selected as heavy label (multiplicity=2). Raw data corresponding to each experiment are reported in Table S1.

## Figures and Tables

**Table 1:** Sample analyzed and experimental design⁎

**Main experiment**	**Species**	**Tissue**	**Type of storage**	**Length of storage**	**Replicates**	**Histone analyzed**	**Histone isolation protocol**	**Super-SILAC spike in**
**Experiment #1**: fresh-frozen and FFPE mouse samples	mouse	spleen	frozen	few weeks	2 biological	H3, H4	#1	no
FFPE	2 biological	#3	no
liver	frozen	few weeks	2 technical	#1	no
FFPE	2 technical	#3	no
spleen	frozen	6 years	2 technical	#1	no
FFPE	2 technical	#3	no
**Experiment #2**: fresh-frozen and FFPE human breast cancer biopsies	human	breast cancer (biopsy #1)	frozen	few months	3 technical	H3	#2	yes
FFPE	3 technical	#3	yes
breast cancer (biopsy #2)	frozen	3 technical	#2	yes
FFPE	3 technical	#3	yes
breast cancer (biopsy #3)	frozen	3 technical	#2	yes
FFPE	3 technical	#3	yes
**Experiment #3**: FFPE human breast cancer biopsies belonging to different subtypes	human	breast cancer - Luminal A	FFPE	few months	5 patients	H3	#3	yes
breast cancer - Luminal B	FFPE	5 patients	#3	yes
breast cancer - Triple Negative	FFPE	5 patients	#3	yes
breast cancer - HER2 positive	FFPE	5 patients	#3	yes

^⁎^ see Table S1 for related raw files.

**Table 2:** Breast cancer biopsies

Breast cancer biopsy #	Subtype^a^	Immunoprofile^b^				pTNM^c^	Grading
ER (%)	PR (%)	HER2	Ki-67 (%)		
1	Luminal B	90	90	60%(+)	25	pT2N1a	G2
2	Luminal A	90	90	–	17	pT1cN0M0	G2
3	Luminal B	50	–	80% (++)	22	pT1cN0M0	G3
1 (LuA)	Luminal A	95	95	–	15	PT1c pNx	G2
2 (LuA)	Luminal A	95	95	–	12	PT2(is) pNza	G2
3 (LuA)	Luminal A	90	90	80% (+)	18	PT2 Pnd (sn)	G2
4 (LuA)	Luminal A	>95	>95	60% (+)	10	PT1a	G1
5 (LuA)	Luminal A	90	80	30% (+)	13	PT2 Pn3a	G2
6 (TN)	Triple Negative	–	–	–	70	PT2 Pn1a	G3
7 (TN)	Triple Negative	–	–	–	75	PT1C Pn0 (sn)	G3
8 (TN)	Triple Negative	–	–	–	60	PT2 Pn1a	G3
9 (TN)	Triple Negative	–	–	–	70	PT2 Pn1a	G3
10 (TN)	Triple Negative	–	–	30% (+)	16	PT1C Pn1mi (sn)	G2
11 (HER)	HER +	–	–	90% (+++)	30	PT1C Pn0 (sn)	G3
12 (HER)	HER +	–	–	>95% (+++)	85	ypT3 ypN2a	
13 (HER)	HER +	–	–	>95% (+++)	46	PT1C Pn0	G3
14 (HER)	HER +	–	–	>95% (+++)	32	rpT1c rpNx	G3
15 (HER)	HER +	–	–	>95% (+++)	75	Pt3 (m) Pn0(sn)	G3
16 (LuB)	Luminal B	95	20	–	45		
17 (LuB)	Luminal B	95	80	70% (++)	55	PT2 (m) Pn1a	G3
18 (LuB)	Luminal B	95	95	65% (+)	25	PT2 (m) PN0	G3
19 (LuB)	Luminal B	95	10	40% (+)	29	Pt1c pNx	G3
20 (LuB)	Luminal B	95	95	60% (+)	26	PT1b Pnd(sn)	G3

a Based on the Ki-67 labeling index value.

b ER: estrogen receptor, PR: progesteron receptor, HER2: human epidermal growth factor receptor 2.

c pTNM: pathological tumor-node-metastasis.

## Footnotes

Appendix A Supplementary data associated with this article can be found in the online version at doi:10.1016/j.dib.2016.02.028.
