# Supplementary material for: Mass-spectrometry analysis of histone post-translational modifications in pathology tissue using the PAT-H-MS approach
Source: Data Brief. 2016 Feb 16;7:188–94. doi: 10.1016/j.dib.2016.02.028 (PMC4927966; doi:10.1016/j.dib.2016.02.028)
Supplement: Supplementary file 1 [file mmc1.docx]

Table S1. Sample analyzed and Experimental design, with reference to the deposited raw files

Main experiment	Species	Tissue	Type of storage	Length of storage	Replicates	Histone analyzed	Histone isolation protocol	Super-SILAC spike in	RAW files
**Experiment #1**: fresh-frozen and FFPE mouse samples	mouse	spleen	frozen	Few weeks	2 biological	H3, H4	#1	no	F140408_RN_SP_H3/H4, F140602_RN_SP_H3/H4
			FFPE		2 biological		#3	no	F140408_RN_FF_H3/H4, F140602_RN_FF_H3/H4
		liver	frozen	Few weeks	2 technical		#1	no	F141028_H3/H4_liver_RN_01-02, F141124_LiverH4RN_1
			FFPE		2 technical		#3	no	F141124_FFH3/H4RN_1-2
		spleen	frozen	6 years	2 technical		#1	no	F141028_H3/H4_spleen_RN_01-02
			FFPE		2 technical		#3	no	F141028_H3/H4_spleenFF_RN_01-02
**Experiment #2**: fresh-frozen and FFPE human breast cancer biopsies	human	breast cancer (biopsy #1)	frozen	few months	3 technical	H3	#2	yes	F140814_Sample2_Frozen_H3_01-03
			FFPE		3 technical		#3	yes	F140814_Sample2_FFPE_H3_01-03
		breast cancer (biopsy #2)	frozen		3 technical		#2	yes	F140814_Sample3_Frozen_H3_01-03
			FFPE		3 technical		#3	yes	F140814_Sample3_FFPE_H3_01-03
		breast cancer (biopsy #3)	frozen		3 technical		#2	yes	F140814_Sample10_Frozen_H3_01-03
			FFPE		3 technical		#3	yes	F140814_Sample10_FFPE_H3_01-03
**Experiment #3**: FFPE human breast cancer biopsies belonging to different subtypes	human	breast cancer - Luminal A	FFPE	few months	5 patients	H3	#3	yes	F150414_RN_H3_1-5
		breast cancer - Luminal B	FFPE		5 patients		#3	yes	F150414_RN_H3_16-20
		breast cancer - Triple Negative	FFPE		5 patients		#3	yes	F150414_RN_H3_6-10
		breast cancer - HER2 positive	FFPE		5 patients		#3	yes	F150414_RN_H3_11-15
